# Blood pressure measurements with the OptiBP smartphone app validated against reference auscultatory measurements

**DOI:** 10.1038/s41598-020-74955-4

**Published:** 2020-10-20

**Authors:** Patrick Schoettker, Jean Degott, Gregory Hofmann, Martin Proença, Guillaume Bonnier, Alia Lemkaddem, Mathieu Lemay, Raoul Schorer, Urvan Christen, Jean-François Knebel, Arlene Wuerzner, Michel Burnier, Gregoire Wuerzner

**Affiliations:** 1 Department of Anesthesiology, Lausanne University Hospital and University of Lausanne, Lausanne, Switzerland; 2 CSEM, Swiss Center for Electronics and Microtechnology, Neuchâtel, Switzerland; 3 Department of Acute Medicine, Geneva University Hospital and University of Geneva, Geneva, Switzerland; 4 Biospectal SA, 1003 Lausanne, Switzerland; 5 Service of Nephrology and Hypertension, Lausanne University Hospital and University of Lausanne, Lausanne, Switzerland

**Keywords:** Diseases, Health care

## Abstract

Mobile health diagnostics have been shown to be effective and scalable for chronic disease detection and management. By maximizing the smartphones’ optics and computational power, they could allow assessment of physiological information from the morphology of pulse waves and thus estimate cuffless blood pressure (BP). We trained the parameters of an existing pulse wave analysis algorithm (oBPM), previously validated in anaesthesia on pulse oximeter signals, by collecting optical signals from 51 patients fingertips via a smartphone while simultaneously acquiring BP measurements through an arterial catheter. We then compared smartphone-based measurements obtained on 50 participants in an ambulatory setting via the OptiBP app against simultaneously acquired auscultatory systolic blood pressure (SBP), diastolic blood pressure (DBP) and mean blood pressure (MBP) measurements. Patients were normotensive (70.0% for SBP versus 61.4% for DBP), hypertensive (17.1% vs. 13.6%) or hypotensive (12.9% vs. 25.0%). The difference in BP (mean ± standard deviation) between both methods were within the ISO 81,060–2:2018 standard for SBP (− 0.7 ± 7.7 mmHg), DBP (− 0.4 ± 4.5 mmHg) and MBP (− 0.6 ± 5.2 mmHg). These results demonstrate that BP can be measured with accuracy at the finger using the OptiBP smartphone app. This may become an important tool to detect hypertension in various settings, for example in low-income countries, where the availability of smartphones is high but access to health care is low.

## Introduction

High blood pressure (BP) remains the leading risk factor for death and disability in both high and low income countries^1^. Its complications are responsible for the deaths of approximately ten million people annually, a 50% increase over the estimates from 1990^2^. By 2025, the number of people suffering from hypertension will reach 1.5 billion^3^. The impact of this disease represents a daunting burden to any healthcare system.

Epidemiological studies have shown that prevalence and control of hypertension plateaued since the mid-2000s in developed countries, while prevalence has increased in developing countries with low rates of awareness and control^4,5^. Early detection and prevention of the complications of hypertension are essential but depend on accessible and accurate measurements. A recent report from the National Health Service (NHS, England) stated that BP from only 60% of registered hypertensive patients was controlled^6^ and only 50% of those starting on a new antihypertensive medications were compliant 6 months later^7^. Issues such as device accessibility, poor patient compliance, physicians’ inertia and the inability of healthcare systems to detect, manage and prevent cardiovascular diseases were among the consequences linked to those findings. The traditional healthcare model of hypertension management based on regular office BP measurements has been challenged, and new hypertension guidelines now strongly recommend the use of home and/or ambulatory BP monitoring for the diagnosis and follow-up of hypertensive patients, thereby motivating and empowering the patient and potentially increasing the frequency of BP measurement^8,9^.

Digital health approaches, and in particular mobile health (mHealth) diagnostics, have been shown to be effective, scalable and sustainable for chronic disease prevention and management^10,11^. Mobile phones represent a widespread, readily available device for mHealth. Worldwide, over one-third of consumers own a mobile phone^12,13^. If the accuracy of reliable smartphone-based blood pressure measurements was to be demonstrated, this would be a promising tool that could improve access to more populations, medical record keeping, analysis of blood pressure measurements for hypertension management as well as medication compliance and health education. Interest in “cuffless” BP measurement, using smartphones or wearable sensor technologies that estimate BP from photoplethysmograms (PPG) is rapidly increasing^14^. In particular, pulse wave analysis techniques^15^, which derive central blood pressure from the morphology of pulse waves in peripheral tissues, are a growing area of interest^16^. Recently, a first wrist-worn device using Pulse Wave Transit Time (PWTT) has obtained a 510(k) clearance by the US Food and Drug Administration for tracking changes in BP following a calibration process using an oscillometric BP monitor (https://www.accessdata.fda.gov/cdrh_docs/pdf19/K190792.pdf, accessed December 10th 2019). However, none of the smartphone-based applications has yet been clinically validated or demonstrated to be accurate^17^.

The aim of this study was to assess the performance of a pulse wave analysis-based BP estimation technique^18^ (oBPM, optical blood pressure monitoring)—previously validated in anaesthesia on pulse oximeter signals^19^—applied to smartphone-derived PPG signals acquired via a dedicated smartphone app (OptiBP). This was accomplished by comparing in an ambulatory setting—using the published ISO 81,060–2:2018 standard^20^—the smartphone-derived BP estimates to simultaneously acquired reference systolic blood pressure (SBP), diastolic blood pressure (DBP) and mean blood pressure (MBP) measurements obtained using the auscultatory method with a dual-head stethoscope.

## Methods

The protocol consisted of two parts: a training part and a validation part. The training part was designed to collect optical signals via the embedded camera of a smartphone running a dedicated mobile app, while simultaneously acquiring invasive intra-arterial BP values. The purpose of this part was to train and further develop an existing algorithm^19^ (oBPM) originally designed to estimate BP values from transmission PPG signals^18^ to reflectance PPG signals acquired by a smartphone camera. The validation part was designed to assess the accuracy of the OptiBP smartphone app on patients using the trained algorithm compared with reference measurements acquired through the classic auscultatory method at the upper arm in an ambulatory setting. This clinical investigation was conducted in compliance with the European Directive 93/42/EEC on medical devices^21^, with international standards ISO 14,155:2011 and the Declaration of Helsinki. The protocol was designed in accordance with the AAMI/ESH/ISO consensus for the validation of BP measuring devices^20^.

### Ethical consent and patient inclusion

The study was approved by the local ethics committee (Commission Cantonale d’éthique de la Recherche sur l’être humain, CH 1012 Lausanne Switzerland, CER-VD no. 2018-01656) and registered under number NCT03875248 at www.clinicaltrials.gov on March 14 2019.

For the training part, written informed consent was obtained from 51 patients aged 18 years or older, who were scheduled for elective surgery necessitating general anaesthesia and invasive arterial BP monitoring at CHUV (University Hospital of Lausanne, Switzerland) or HUG (Geneva University Hospital). Exclusion criteria were patient refusal, arrhythmia and inability to give informed consent.

For the validation part, written informed consent was obtained from 50 participants (hypertensive or not) aged 18 years or older and scheduled for an elective visit at the outpatient hypertension clinic at CHUV (University Hospital of Lausanne, Switzerland). Exclusion criteria were patient refusal, inability to give informed consent, dysrhythmia or an arterial disease leading to a SBP difference > 15 mmHg or DBP difference > 10 mmHg between arms^20^.

### Methodology for training OptiBP

#### Anesthesia protocol and signal acquisition

**Figure 1:**
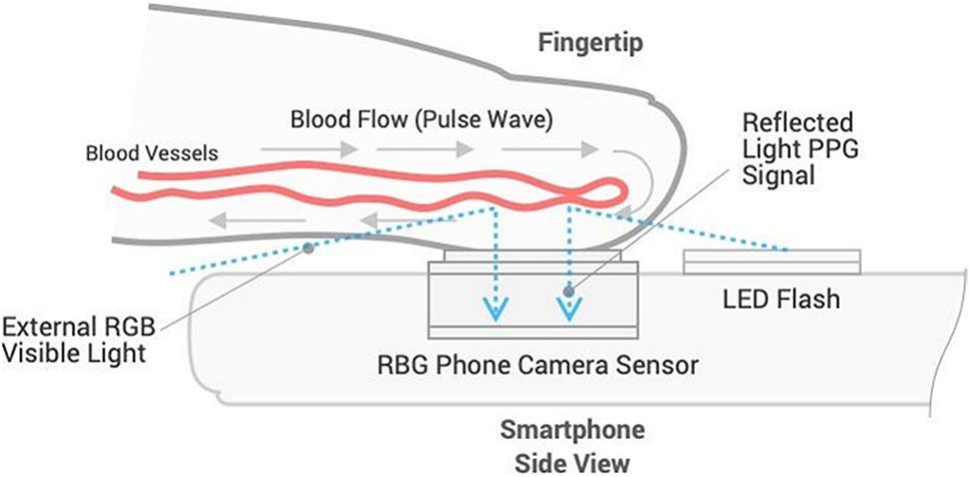
OptiBP application utilizes image data generated from volumetric blood flow changes via light passing through the fingertip, reflecting off of the tissue, and then passing to the phone camera's image sensor.

On the day of surgery, patients were monitored according to the standards of our departments and connected to a Philips IntelliVue MP50 monitor (Philips, Amsterdam, the Netherlands). A 20-gauge, 4.5 cm length arterial catheter (BD FlowswitchTM, Becton–Dickinson, Franklin Lakes, USA) was inserted under local anaesthesia predominantly into the left or right radial artery. The transducer for the intra-arterial catheter was kept at the level of the left ventricle of the heart. A flush test was performed to rule out over- or under-damping^22^. The continuous invasive SBP, DBP, and MBP, were recorded before and during induction of general anaesthesia for one minute followed by an average per-patient duration of 20 min with the ixTrend express software version 2.1.0 (ixellence GmbH, Wildau, Germany) installed on a laptop computer connected to the monitor. Each minute of recording generated one average BP value. Simultaneously, a customized app installed on a Samsung Galaxy S7 (Samsung GEC, 26, Sangil-ro 6-gil, Gagdong-gu, Seoul, Korea) recorded a raw optical signal consisting of a sequence of images at 30 Hz. Each session encompassed 10 series of recordings of 1 min, each separated by a 1-min interval free of recording. The procedure included the positioning of the patient’s middle or index finger on the phone camera lens (Fig. 1) and launching the application at the beginning of the protocol. A timestamp allowed simultaneous correlation of the smartphone acquired signals with the invasive measurements. General anaesthesia was induced and maintained with an infusion of propofol while intravenous analgesia was provided by boluses of fentanyl or continuous remifentanil, depending on the surgical intervention. The management of the anaesthesia was at the discretion of the anaesthesiologist.

#### Smartphone data processing and analysis

**Figure 2:**
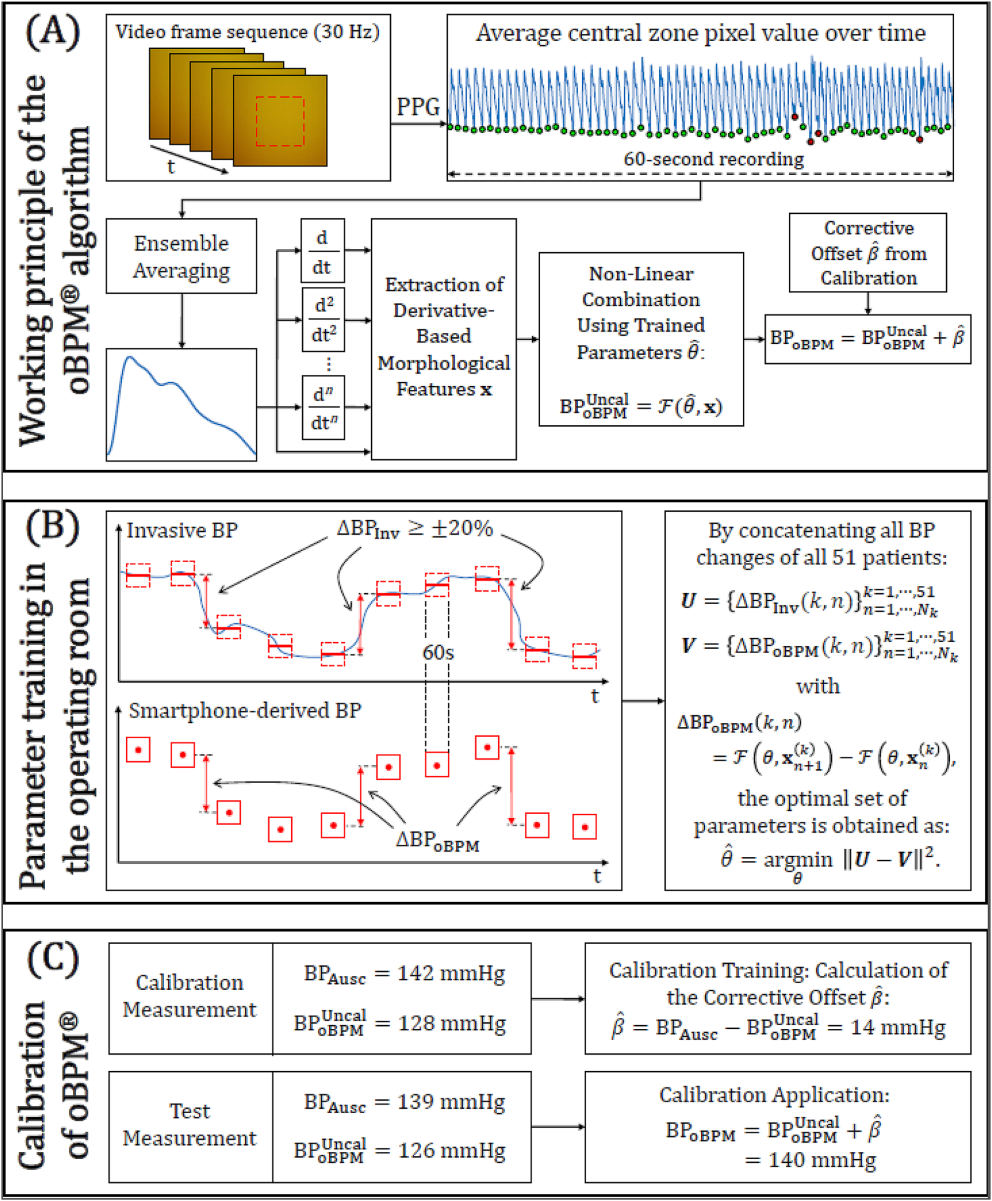
Algorithm description, parameter training, and calibration. (**A**) Working principle of the oBPM (optical blood pressure monitoring) algorithm. The oBPM algorithm automatically identifies all individual pulses in the PPG signal and ensemble averages them. Pulses with un-physiological morphologies (red dots) are identified and assigned low weights in the ensemble averaging procedure, whereas the remaining pulses (green dots) are assigned a stronger influence. The resulting ensemble average waveform is fed to a filter bank of time-derivative filters, allowing a decomposition of the waveform at various time resolutions. From their outputs, a set of features $\mathbf{x}$ characterizing the morphology of the waveform is obtained and nonlinearly combined using a pre-trained set of parameters $\widehat{\theta }$ (see (**B**) panel of the figure). The result is an uncalibrated BP value, ${\mathrm{BP}}_{\mathrm{oBPM}}^{\mathrm{Uncal}}$. The final oBPM-derived BP estimate (${\mathrm{BP}}_{\mathrm{oBPM}}$) is obtained after application of the previously trained corrective calibration offset $\widehat{\beta }$. (**B**) Training of the parameters of the oBPM algorithm. The parameters were trained using the data acquired in the operating room. Significant BP changes ($\Delta {\mathrm{BP}}_{\mathrm{Inv}}\ge \pm 20\mathrm{\%}$) between successive recordings were identified in the arterial line measurements. Their corresponding oBPM-derived BP changes ($\Delta {\mathrm{BP}}_{\mathrm{oBPM}}$) were then calculated to be compared. The set of oBPM parameters $\theta$ was optimized by minimizing the cohort-wise error between $\Delta {\mathrm{BP}}_{\mathrm{oBPM}}$ and $\Delta {\mathrm{BP}}_{\mathrm{Inv}}$ in the least-square sense. In the figure, ${N}_{k}$ is the number of significant BP changes found for patient $k$, and $\mathcal{F}$ is the non-linear oBPM model mapping the features $\mathbf{x}$ to BP values using the parameters $\theta$. (**C**) Illustration of the calibration procedure. The calibration consists in the addition of a per-patient corrective offset $\widehat{\beta }$ to the uncalibrated oBPM-derived BP estimate ${\mathrm{BP}}_{\mathrm{oBPM}}^{\mathrm{Uncal}}$ for systolic, diastolic and mean BP individually. It is illustrated here with numerical values for ease of understanding. During the calibration measurement, the corrective offset $\widehat{\beta }$ is calculated. Applying the calibration to the following test measurements consists of the addition of $\widehat{\beta }$ to the uncalibrated BP estimate outputted by oBPM.

All smartphone data were analysed and post-processed offline using MATLAB (The MathWorks, Inc., Natick, USA). For each 1-min sequence of images acquired with the smartphone, a BP estimate (${\mathrm{BP}}_{\mathrm{oBPM}}$) was obtained in a fully automated manner via the oBPM algorithm^18^ as illustrated in Fig. 2A. As a first step, the pixels from the green channel of the central region of each image in the video sequence were averaged to obtain a PPG signal. Each pulse in the 1-min PPG signal was given a quality index according to its similarity to its neighbouring pulses, resulting in less weight given to pulses with aberrant morphologies. All pulses were then averaged to obtain a representative pulse wave shape. An acceptance criterion based on the average quality of the pulses and the waveform of the averaged pulse was computed, which allowed automatic rejection of average pulses with un-physiological morphology or those obtained from PPG signals with low quality pulses (e.g. in cases of excessive finger movement, poor positioning or excessive finger-camera pressure). Each accepted average waveform was passed through a bank of time-derivative filters, which allowed characterization of the morphological variations of the pulse at various time resolutions. A set of derivative-based features $\mathbf{x}$ was extracted and non-linearly combined to obtain an uncalibrated estimate of BP, referred to as ${\mathrm{BP}}_{\mathrm{oBPM}}^{\mathrm{Uncal}}$. The oBPM algorithm can provide absolute values of BP changes around an arbitrary baseline value, but requires an initial calibration procedure to set this baseline value and thereby provide absolute BP estimates. Therefore, obtaining the final calibrated oBPM-derived BP estimate ${\mathrm{BP}}_{\mathrm{oBPM}}$ required applying a corrective calibration offset $\widehat{\beta }$, such that ${\mathrm{BP}}_{\mathrm{oBPM}}={\mathrm{BP}}_{\mathrm{oBPM}}^{\mathrm{Uncal}}+\widehat{\beta }$. This calibration procedure will be further detailed in “Smartphone data processing and analysis”

#### Algorithm parameter training

The parameters $\widehat{\theta }$ of the non-linear model applied to the feature set $\mathbf{x}$ generated by the oBPM algorithm were trained using the data from the 51 patients obtained in the operating theatre OptiBP. This process is illustrated in Fig. 2B. Following ten 1-min recordings per patient, nine pairs of successive measurements were obtained for each patient. All pairs showing a significant change in BP in the invasive reference (${\mathrm{BP}}_{\mathrm{Inv}}$) data were selected. A change of at least 20% was considered significant^23^ ($\Delta {\mathrm{BP}}_{\mathrm{Inv}}\left(n\right)\ge \pm 20 \%$, with $\Delta {\mathrm{BP}}_{\mathrm{Inv}}\left(n\right)=100 \% \cdot  \left[{\mathrm{BP}}_{\mathrm{Inv}}\left(n+1\right)-{\mathrm{BP}}_{\mathrm{Inv}}\left(n\right)\right]/ {\mathrm{BP}}_{\mathrm{Inv}}(n))$. Their corresponding oBPM-derived BP changes ($\Delta {\mathrm{BP}}_{\mathrm{oBPM}}$) were selected as well. By concatenating all selected BP changes $\Delta {\mathrm{BP}}_{\mathrm{Inv}}$ and their corresponding $\Delta {\mathrm{BP}}_{\mathrm{oBPM}}$ for all patients in vectors ${\varvec{U}}$ and ${\varvec{V}}$ respectively, the parameters $\widehat{\theta }$ of the model were optimized in a least-square sense, *i.e.* by solving $\widehat{\theta }={\underset{\theta }{\mathrm{argmin}} \Vert {\varvec{U}}-{\varvec{V}}\Vert }^{2}$. The thus-trained model was then applied to the validation dataset of the hypertensive unit OptiBP part.

### Methodology for validating OptiBP

#### Hypertension unit protocol

For each measurement, auscultatory BP values were measured and optical signals were acquired simultaneously using the reference device on one arm and the test device on the fingertip of the opposite arm (same Samsung Galaxy S7 phone as for the training OptiBP part, which acquired the same raw optical signal using the same proprietary app). The reference device was a validated non-invasive (auscultatory) sphygmomanometer (A&D UM-101, A&D Company, Ltd., Toshima Ku, Tokyo, Japan), which was calibrated before the study, allowing intermittent BP measurements operated by two independent trained observers using a dual-head stethoscope. The two healthcare professionals formed a trained team that was validated before the onset of the study. The ISO specification requires the two observers to have a maximal difference in the measure of systolic and diastolic BP of 4 mmHg and successive recordings to have variations of at most 12 mmHg SBP and 8 mmHg DBP. In case of failure of the above, the measurements were discarded. The protocol included 7 sequential measurements of BP every 2 min. After four valid measurements, the devices were interchanged. The starting arm side for the reference reading was alternated between subjects: odd-numbered subjects started with the reference device on the right arm and even-numbered subjects on the left arm (Table 1).

**Table 1:** Study protocol for patients included in the validation set.

**Subject preparation**
At least 5 min relaxed in an isolated and quiet room at a comfortable temperature Back, elbow and forearm supported, legs uncrossed, and feet flat on the floor, empty bladder Appropriate cuff size and smartphone at the level of the left ventricle of the heart
**Lateral difference determination (LD)**
3 reference measurements on right arm 3 reference measurements on left arm
**Simultaneous OptiBP and Reference BP recordings**
Measurements #0 (for screening and calibration, OptiBP on right arm for even-numbered subject and left for uneven) Reference measurement (R0) and OptiBP measurement (T0) Wait at least 60 s
Measurement #1 Reference measurement (R1) and OptiBP measurement (T1) Wait at least 60 s
Measurement #2 Reference measurement (R2) and OptiBP measurement (T2) Wait at least 60 s
Measurement #3 Reference measurement (R3) and OptiBP measurement (T3) Wait at least 60 s and interchange arm sides
Measurement #4 (for calibration) Reference measurement (R4) and OptiBP measurement (T4) Wait at least 60 s
Measurement #5 Reference measurement (R5) and OptiBP measurement (T5) Wait at least 60 s
Measurement #6 Reference measurement (R6) and OptiBP measurement (T6) Wait at least 60 s
Extra measurement #7 Reference measurement (R7) and OptiBP measurement (T7) Wait at least 60 s
Extra measurement #8 Reference measurement (R8) and OptiBP measurement (T8)

*R* reference measurement, *T* tested device measurement (OptiBP).

The protocol was overseen by a supervisor who monitored the adequacy of reference and test device BP measurements, the agreement between the two observers, who were unaware of the magnitude or direction of their disagreement, and any other issue arising during the validation procedure. The supervisor reviewed each pair of test/reference BP measurements.

For both study parts, demographic values such as sex, age, height, weight and stage of hypertensive disease were documented, as well as optical signals, references and estimated BP values.

#### Smartphone data processing and analysis

The image acquisition by the smartphone and the post-processing of its data via the oBPM algorithm were identical to the training OptiBP part. The trained algorithm was applied as is on the data of the validation OptiBP part. Unreliable estimates were automatically rejected by the oBPM algorithm. A calibration procedure was then applied as explained thereafter and illustrated in Fig. 2C. The corrective offset $\widehat{\beta }$ is computed during the calibration measurement for each patient by comparing the oBPM-derived BP estimate to its corresponding auscultatory-derived value and applying this correction to all following BP estimates. According to wave reflection theory^24^, the morphology of the waveform could be different from one arm to another as the path taken by the pulse is different, thus the calibration procedure was performed separately on each arm.

### Statistical analysis

The accuracy of our approach to estimate absolute BP values was assessed by comparing the oBPM-derived SBP (SBP_oBPM_), DBP (DBP_oBPM_) and MBP (MBP_oBPM_) estimates to their corresponding auscultatory-derived reference values (SBP_Ausc_, DBP_Ausc_, MBP_Ausc_). MBP_Ausc_ was estimated as 2/3 DBP_Ausc_ + 1/3 SBP_Ausc_. We computed the mean and standard deviation (SD) of the estimation error both at a cohort-wise and patient-wise level to determine group and within-patient accuracy. We also computed the percentage errors in order to assess the performance of each pressure measurement (systolic, diastolic, and mean) independently of their absolute values. In the absence of standards applicable to our cuffless approach, we evaluated the performance of the smartphone-based BP estimations in an ambulatory setting using the standards of the ISO 81060–2:2018 norm^25^. This standard requires the mean value of the errors of the individual paired determinations of the device-under-test to be less than or equal to ± 5.0 mmHg with an experimental standard deviation (SD) no greater than 8.0 mmHg.

## Results

### Dataset for training OptiBP

The demographics of the 51 patients used for training the algorithm are shown in Table 2 for the 26 males and 25 females. One patient had to be excluded due to a failed attempt to cannulate the radial artery. The distribution of the reference systolic BP training set consisted of 53 recordings (18.0%) higher than 140 mmHg, 148 (50.4%) between 100 to 140 mmHg and 93 (31.6%) below 100 mmHg while diastolic BP was higher than 80 mmHg in 19 (6.4%) recordings, between 60 to 80 mmHg in 111 (37.8%) and below 60 mmHg in 164 (55.8%) recordings.

**Table 2:** Participants’ characteristics (n = 51) used to train oBPM algorithm.

	Mean	SD	Range
Age (years)	61·6	13·5	24·0–87·0
Height (cm)	170·1	9·0	143·0–190·0
Weight (kg)	77·9	18·9	40·0–139·0
BMI (kg/m^2^)	26·8	6·0	26·4–46·9

### Dataset for validating OptiBP

We report on the first 50 patients that were included between July 1 and September 30 2019 in the hypertensive unit validation part, including 16 males and 24 females. Data from 40 patients generated an adequate signal and allowed smartphone BP measurements. Their demographics and specifics are shown in Table 3. Hypertension had been diagnosed in 15 patients (37.5%) and qualified as pre-hypertension in 2 patients (13.3%), stage 1 hypertension in 7 (46.7%), stage 2 in 5 (33.3%) and stage 3 hypertension in 1 patient (6.7%). Dedicated hypertensive treatment was ongoing in 13 patients (86.7%).

**Table 3:** Participants’ characteristics (n = 40) used to validate OptiBP.

	Mean	SD	Range
Age (years)	53·9	17·5	21·0–78·0
Height (cm)	169·4	10·6	154·0–198·0
Weight (kg)	73·4	20·4	45·0–152·0
BMI (kg/m^2^)	25·3	5·2	18·0–44·6
Systolic BP (mmHg)	121·0	18·7	84·2–163·3
Diastolic BP (mmHg)	75·0	11·7	51·2–103·3
Mean arterial BP (mmHg)	90·3	12·1	65·1–119·9

Table 4 shows the distribution of their cuff-based blood pressure recordings with a majority of the blood pressure values within the non-hypertensive range (< 140/90 mmHg).

**Table 4:** Distribution of reference systolic and diastolic blood pressure (BP) measurements (n = 140) in OptiBP validation population.

	N	%
**Systolic BP**		
High BP (SBP ≥ 140 mmHg)	24	17·1
Normal BP (100 mmHg ≤ SBP < 140 mmHg	98	70·0
Low BP (SBP < 100 mmHg)	18	12·9
**Diastolic BP**		
High BP (DBP ≥ 90 mmHg)	19	13·6
Normal BP (65 mmHg ≤ DBP < 90 mmHg)	86	61·4
Low BP (DBP < 65 mmHg)	35	25·0

**Figure 3:**
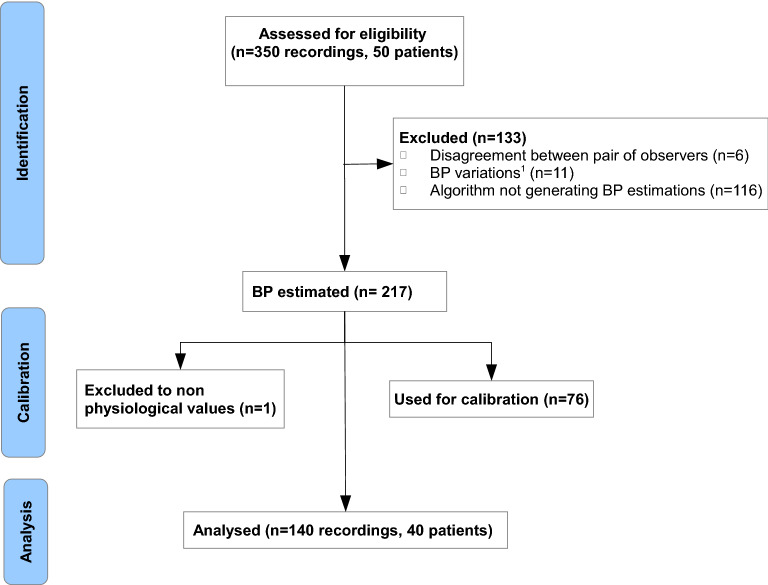
CONSORT Flow Chart of signals used for validation. ^1^SAP > 12 mmHg or DAP > 8 mmHg.

Of the 350 signals recorded (see Fig. 3 consort flow diagram), 133 were not eligible due to non ISO conformity (17) or technical issues (116) such as image instability related to excessive finger movement or pressure.

Forty patients generated 217 recordings, of which 76 were used to calibrate the optical signal and therefore were excluded from the analysis to avoid overfitting. One additional recording generated a non-physiological value and was automatically rejected.

### Accuracy of OptiBP

A total of 140 BP values were generated with an overall performance of the smartphone blood pressure measurement compared to the manual auscultatory device detailed in Table 5 for the systolic, diastolic and mean arterial pressure.

**Table 5:** Overall performance of OptiBP compared to dual-head stethoscope cuff measure.

	Mean error		SD	
	mmHg	%	mmHg	%
Systolic BP	− 0·7	− 0·6	7·7	6·7
Diastolic BP	− 0·4	− 0·5	4·5	6·5
Mean BP	− 0·6	− 0·6	5·2	6·1

**Figure 4:**
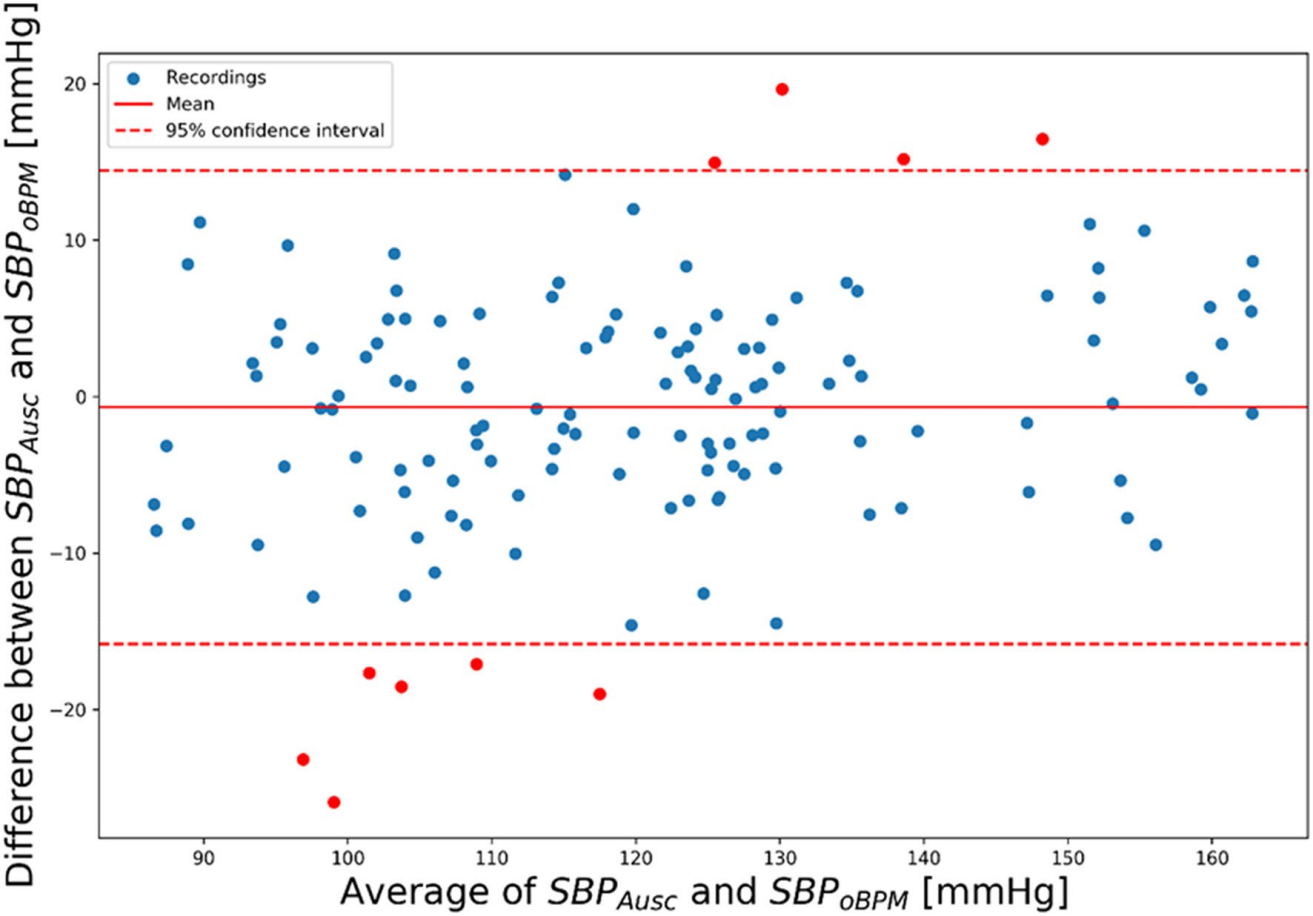
Standardized Bland–Altman scatterplots depicting the agreement between the OptiBP smartphone app systolic estimates assessed by the oBPM algorithm (SBP_oBPM_) and the auscultatory-derived reference systolic BP measurements (SBP_Ausc_).

**Figure 5:**
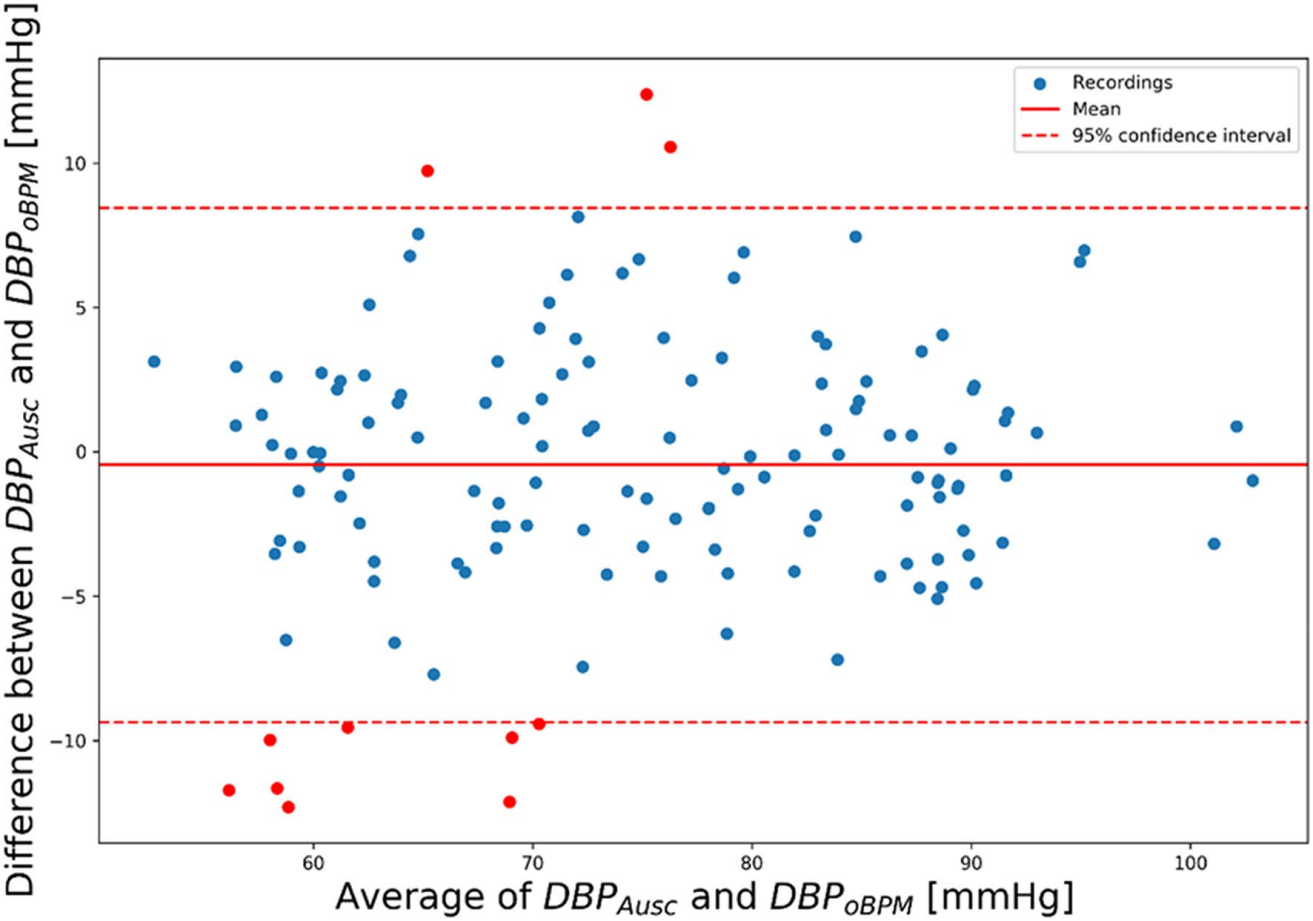
Standardized Bland–Altman scatterplots depicting the agreement between the OptiBP smartphone app diastolic estimates assessed by the oBPM algorithm (DBP_oBPM_) and the auscultatory-derived reference systolic BP measurements (DBP_Ausc_).

**Figure 6:**
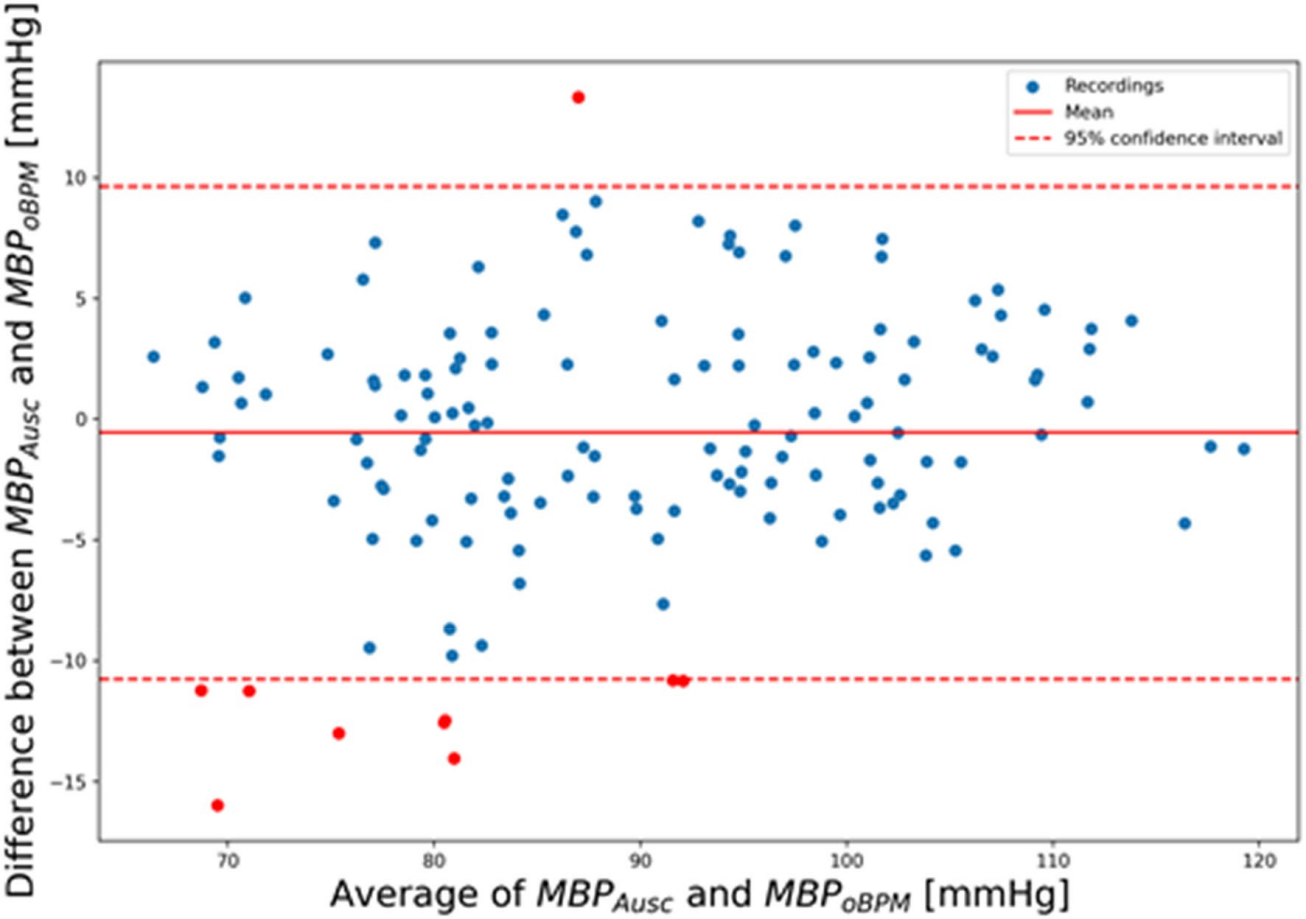
Standardized Bland–Altman scatterplots depicting the agreement between the OptiBP smartphone app mean BP estimates assessed by the oBPM algorithm (MBP_oBPM_) and the auscultatory-derived reference systolic BP measurements (MBP_Ausc_).

The mean and SD of the error were of − 0.7 ± 7.7 mmHg for SBP, − 0.4 ± 4.5 mmHg for DBP and − 0.6 ± 5.2 mmHg for MBP and complied with the requirements of the ISO81060-2:2018 norm. Standardized Bland–Altman scatterplots are shown in Figs. 4, 5 and 6 for SBP, DBP, and MBP, respectively. The solid line represents the mean error (bias), whereas the dotted lines indicate the 95% confidence interval (mean ± 1.96 SD).

## Discussion

This study shows that our smartphone-based optical blood pressure measuring algorithm is accurate when compared to the reference auscultatory method in an office-based setting.

To the best of our knowledge, our smartphone OptiBP app is the first to fulfil the criteria of the ISO81060-2:2018 norm designed to validate cuff-based intermittent blood pressure measurements, without the need for additional hardware. Instant blood pressure (IBP; AuraLife, Newport Beach, CA) was a BP-measuring app that was available on the iTunes store from June 2014 through July 2015 and claimed to estimate BP by placing the smartphone on the chest while the user placed his right index finger over the smartphone’s camera. The BP measurements of this popular app was demonstrated as highly inaccurate with a mean (standard deviation) absolute difference between IBP and the reference device to be 12.4 (10.5) mmHg for systolic BP and 10.1 (8.1) mmHg for diastolic BP^17^. Following this publication, the Federal Trade Commission pursued litigation against AuraLife and the app was removed from iTunes.

Kuwabara et al.^26^ demonstrated that a watch-based wearable monitor, consisting of an oscillometric cuff-based automatic BP monitor integrated in a wrist band, fulfilled the ISO81060-2:2013 validation criteria in a standard sitting position with the watch at heart level. Its small size and silent measurement functions were interesting advantages compared to existing cuff devices and would allow access to measure BP in specific situations and settings^27^. However, due to the automatic cuff-based BP measurement, the accuracy under real-world conditions and how it might be affected by body, palm position or wrist size must be assessed. Furthermore, it requires the acquisition of a specific device and might represent similar burdens as the automatic cuff, including discomfort and occlusive blood pressure measurement.

Watanabe et al.^28^ described a cuffless BP estimation device including a photoplethysmographic sensor, coupled to an algorithmic transformation of the optical signal, and used it on 35 patients. Changes in light absorption at the participant’s right index correlated to changes in peripheral blood volume and were transformed by their algorithm into BP estimates after initial calibration. Although they were able to demonstrate agreement of their sensor assemblies coupled to an algorithm with standard cuff-based measurements under static and dynamic conditions, the applicability to a commercially available device is not yet realized and reproducibility has yet to be established in various settings. Based on the measurement of a pulse transit time from an electrocardiogram and a PPG signal at the fingertip, the Checkme Pro Health Monitor (Shenzen Viatom, China) has been compared with the measurement of SBP from a classic home BP monitor^29^. Although the device was complex to use and the success rate of the SBP measurement was low, it is noteworthy that participants indicated an increased willingness to take their BP measurement. The small study sample size and the restriction to the sole value of SBP limits its validity, particularly in young hypertensive patients^30^.

Transdermal optical imaging (TOI) allowing detection of facial blood flow changes with a smartphone camera in combination with advanced machine learning techniques has been shown to be capable of determining BP values^31^. This technology uses state-of-the-art remote PPG^32^ with extractions of raw signals and estimation of plethysmography signal (band pass filtering). Using a machine learning-based algorithm from a FDA-cleared BP measurement system, TOI extracts haemoglobin-rich signals that are recombined and linked to represent facial blood flow oscillations. BP was measured on normotensive patients in an experimental setting after an individual calibration. The system needs to be validated with changing blood pressures before its usefulness can be assessed.

Our research protocol was designed according to the ISO81060:2-2018 norm and conducted in an ambulatory setting in a leading Swiss hypertension University clinic by specifically trained personnel on 50 patients. The parameters of our oBPM algorithm were trained in challenging operating room conditions (large BP variations) against an invasive reference, thereby ensuring its sensitivity to such changes. Our results demonstrate that a smartphone-model unveiled 4 years ago, without any additional hardware, can be used to measure BP accurately with 70% of the optical signals of sufficient quality to be interpreted. While no cut-off values have been defined to declare the device reliable, future improvement in technology and signal processing will further increase the acceptance rate. Our results suggest that smartphones offer a high potential to improve the accessibility of BP measurements to a broad population, since there is deep penetration of such devices in almost all geographical areas of the world^33^. The public’s interest in downloading smartphone applications for hypertension management is suggested by the fact that some apps for BP measurement, despite not disclosing the methodology nor the accuracy of the measurements, were downloaded more than one million times in 2015^34^.

Although the prevalence of hypertension in developed countries has remained stable in recent years, its increase in low- and middle-income countries represents a future burden to any healthcare system. In many parts of the world, health care services are limited and can provide only occasional and unstructured care for blood pressure related diseases. Cuffless blood pressure measurement is receiving increased attention since it has the potential to overcome some of the shortcomings related to cuff based blood pressure measurements, mainly accessibility of the device^35^. Smartphones and mHealth could play a promising role in both high-income and developing countries to reduce the adverse effects of hypertension^33^, by maximizing the synergies of mHealth and eHealth as demonstrated in various settings^36^.

Finally, although no universally accepted standards for the validation of the cuffless BP measurement currently exist, groups of experts have participated in the development of International standards^37^. In 2018, the Association for the Advancement of Medical Instrumentation (AAMI), the European Society of Hypertension (ESH) working group on BP monitoring and the International Organization for Standardization (ISO) presented principles and key elements of a universal validation procedure. With this publication, the basis to enforce regulatory requirements over apps that diagnose, treat or prevent a medical condition may become clearer^38^. The existing IEEE Std 1708 establishes a normative definition of wearable cuffless blood pressure measuring devices and the objective performance evaluation of such devices^39^. While this is not a fundamental deficiency of new systems and this only reflects the “first-to-market” advantage of cuff devices, issues in practicality and maintenance remain to be studied regarding oBPM technology.

### Limitations

In the current study, we present the preliminary blood pressure results based on the analysis of the first 50 patients. Further data collection is ongoing and additional work needs to be conducted on various subpopulations and larger groups of patients where accuracy might be different. Furthermore, in our study, the population recruited so far does not correspond to the distribution of blood pressure values requested by the ISO standards.

A second seeming limitation is that a calibration procedure was established for every patient individually in our study, as could be happening at a clinic or at the patients’ general practitioner. A preliminary longitudinal study in healthy subjects was able to demonstrate calibration stability for 3 months with a standard deviation of 8% on systolic and diastolic BP^40^. A similar longitudinal study must now validate those findings in a hypertensive population. The cuff-based devices that have been validated using the static ISO norms are based on the assumption that the rested device is capable of measuring BP without an initial calibration reference measurement. In real life, a cuffless BP monitor should operate accurately over a range of pressure after being calibrated at a known pressure. Further explorations related to the cuffless-BP measure must be undertaken to better report specifics related to this new technology.

## Conclusion

This study demonstrates the high accuracy of the OptiBP smartphone app to estimate blood pressure in a subset of 40 patients with a mean and SD of the error of − 0.7 ± 7.7 mmHg for SBP, − 0.4 ± 4.5 mmHg for DBP and − 0.6 ± 5.2 mmHg for MBP and complied with the requirements of the ISO81060-2:2018 norm. This widely-available device could impact health assessment capabilities in developed and third world countries.
